# Brittle Platinum Pins in Porcelain Teeth

**Published:** 1899-11

**Authors:** George M. P. Murray

**Affiliations:** Dublin.


AMERICAN JOURNAL

—OF—

DENTAL SCIENCE.

VOL. XXXIII. BALTIMORE, NOVEMBER, 1899. NO. 7.

### Selections.

ARTICLE I.

### BRITTLE PLATINUM PINS IN PORCELAIN TEETH.

GEORGE M. P. MURRAY, F. R. C. S., DUBLIN.

As compared with the delicate spectroscopic analysis of Professor Hartley, the experiments carried out by us are, perhaps, crude and somewhat "rough-and-ready," but the "dropping off" of teeth in practice is a "rough-and ready" affair, and the language used by patients in describing it is sometimes rather "rough-and-ready," too. We may, at all events, claim for these experiments the merit of being practical, and of serving to eliminate some of the suggested causes of this mishap.

Before proceeding to a detailed description of these tests, it may be well to impress once more upon all interested in the matter that our object has not been to fix blame upon anyone, but simply to find out the truth, and the object of the investigation would be just as fully and satisfactorily achieved if we could prove to demonstration that these fractures were due to some faulty procedure on the dentist's part, as if we were to find a definite and damning impurity in the pins of the teeth as supplied to us by the manufacturers. To find the true cause and its remedy has been, then, our sole object.

The negative result of Professor Hartley's spectrum analysis cuts two ways. It proves that the brittle pins contained originally no metallic impurity; but it also proves that no such impurity was introduced by the dentist's fault, either by the use of base or unsuitable solder, contaminated investment, or in any other way.

It is probably the experience of most victims of this very annoying and discerning accident that it is more often found to happen with plate teeth than with vulcanite, but this, we venture to submit, does not prove that soldering has anything whatever to do with the case, or that brittle pins exist more frequently in teeth so dealt with.

The brittle pins have some cohesion and some tensile resistance is, we think, evident from the fact that they often stand use for some weeks before parting company with their mounting; and it would seem as if they had a better chance of surviving when anchored into a comparatively yielding and elastic material like vulcanite, than when held between two such rigid substances as the porcelain and a metal backing—a condition specially unfavorable for the withstanding by brittle crystalline metal of even a slight jar. The fact of the lesion ever occurring in connection with vulcanite proves far more in one direction than the fact of its happening perhaps less frequently can possibly be held to prove in the other.

My first experience of "dropped-off" teeth was with four bicuspids which quietly came away from a vulcanite mauillary denture.

Engineers tell us of some conditions under which steel becomes brittle and unreliable, but that is after prolonged strain and constant vibration, whilst the fracture under consideration invariably occurs at the very outset of the pin's promising career, and in no case has it happened to teeth which had been long in use.

I would venture to anticipate a suggestion which has not yet been advanced, but will probably occur to some one to bring forward as a simple cause—namely, that a faulty backing, not properly home to the porcelain, throws a heavy strain upon the pins at their point of exit from the tooth, whilst in vulcanite teeth it might be held that insufficient support in the rubber threw all the strain upon the same point. These hypotheses, however, are exploded by one of Professor Hartley's experiments, in which he demonstrates that the fragments of the broken pins are themselves brittle and crystalline, and when fused into a button the metal still retained these defects, thus showing that the brittleness was not confined to one point.

Light is thrown on an investigation of this kind by spontaneous and by deliberate experiments. Under the former heading falls our observation of what occurs in the ordinary course of things. Thus we observe that the heat applied to platinum in making the pins does not ordinarily render it brittle, neither does the heat of baking the porcelain. If heat were the cause, all the pins should be brittle. Nor are cases wanting, as we have seen, where the pins break without being subjected to any high temperature in soldering. We also know from observation, or spontaneous experiment, that direct violence usually breaks the porcelain, leaving the pins intact, and that a prolonged strain will draw the pins out of vulcanite, or even stretch them.

These observations prove much, but we have thought it well to supplement them by certain deliberately applied tests under conditions carefully noted and controlled.

### NOTES ON SOME EXPERIMENTS WITH PLATINUM PINS.

For experimental purposes fourteen plate teeth were taken, with presumably tough pins, as they had been in use in the mouth for over ten years—viz., nine American teeth (gold backing), and five English teeth (D. A. backing). All had been riveted.

No. 1. With knife and hammer an attempt was made to split off English tooth from backing. Result: Tooth broke away, leaving pins intact in backing.

No. 2. American tooth, similarly treated. Result: Identical with No. 1.

No. 3. American tooth—(a) similarly treated. Result: Same as foregoing (considerable force required to split tooth away). (b) From the backing one of the pins was broken away with pliers. Fracture fibrous.

These three experiments were intended to test toughness of pins in the teeth selected for experiment.

No. 4. American tooth invested in plaster and fresh sand, dried out and heated with blowpipe till backing was fused, then split off with knife and hammer. Result: Pins so soft that knife cut deeply into them, one breaking through the uncut portion with fibrous fracture, the other coming away with backing, the latter, when broken off with pliers, shows fibrous fracture also.

No. 5. A repetition of No. 4. Result: Pins remained intact in backing, one being broken with pliers shows fibrous fracture.

No. 6. English tooth, invested in plaster and sand, containing fillings of lead and zinc. Heated up and soldered, and split away with knife and hammer. Result: Very difficult to split off. Pins remained intact in backing, tooth breaking away. One pin being broken off with pliers, shows fibrous fracture.

No. 7. A repetition of No. 6. Result: Identical.

No. 8, American tooth. Investment containing lead and zinc fillings. Heated to the extent of fusing backing, and then split off as in other cases. Result: Knife nearly cut through pins, fracture of uncut portion being fibrous

No. 9. English tooth, invested in plaster and fresh sand, heated up and soldered with smoky flame, and split off as in other cases. Result: Pins cut through by knife.

No. 10. English tooth similarly treated. Result: One pin broke (fibrous fracture) the other coming away intact with backing.

No. 11. American tooth. Investment containing zinc and lead fillings, heated up with smoky flame till backing was fused, split off as before. Result: One pin nearly cut through, fracture through remaining portion fibrous. Other pin came away with backing.

No. 12. American tooth, invested in plaster and clean sand. Heated up and soldered with smoky flame, and split away as in other cases. Result: Pins remained intact in backing.

No. 13. A repetition ot No. 12. Result: Pins very soft, nearly cut through.

No. 14. A repetition of No. 12. Result: Pins came away with backing; one, on being broken with pliers, shows fibrous fracture.

These experiments would seem to show that neither overheating, "impurities in investment," nor "a smoky flame," will cause sound platinum pins to become brittle or to break with the peculiar crystalline fracture which characterises the pins of the teeth which "drop off."

FURTHER EXPERIMENTS.

Four unusual American teeth were taken, the remainder of a set of six. Of the two used, one had "dropped off" after a few days' use; the other is in use a year.

No. 15. The pins of three of these teeth were broken with pliers, leaving a fibrous fracture.—*Ia. Dental Journal.*

